# Lumpy skin disease in Kazakhstan

**DOI:** 10.1007/s11250-021-02613-6

**Published:** 2021-02-15

**Authors:** Mukhit B. Orynbayev, Raikhan K. Nissanova, Berik M. Khairullin, Arman Issimov, Kunsulu D. Zakarya, Kulyaisan T. Sultankulova, Lespek B. Kutumbetov, Ali B. Tulendibayev, Balzhan Sh. Myrzakhmetova, Erbol D. Burashev, Sergazy S. Nurabayev, Olga V. Chervyakova, Aziz K. Nakhanov, Richard A. Kock

**Affiliations:** 1 RGE ‘Research Institute for Biological Safety Problems’, Committee of Science, The Ministry of Education and Science of the Republic of Kazakhstan, Gvardeiskiy, Zhambyl Region Republic of Kazakhstan; 2 Kazakh National Agrarian University, Almaty, 050010 Republic of Kazakhstan; 3 Kyrgyz National Agrarian University named after K.I.Skryabin, Bishkek, Kyrgyzstan; 4 Sydney School of Veterinary Science, Faculty of Science, University of Sydney, Sydney, Australia; 5 Pathobiology and Population Sciences, Royal Veterinary College, Hawkshead Lane Herts, AL9 7TA UK

**Keywords:** Lumpy skin disease, Kazakhstan, Vector-borne disease, Tick, Insect

## Abstract

This study describes the registration of the first cases of lumpy skin disease in July 2016 in the Republic of Kazakhstan. In the rural district of Makash, Kurmangazinsky district of Atyrau region, 459 cattle fell ill and 34 died (morbidity 12.9% and mortality 0.96%). To determine the cause of the disease, samples were taken from sick and dead animals, as well as from insects and ticks. LSDV DNA was detected by PCR in all samples from dead animals and ticks (Dermacentor marginatus and Hyalomma asiaticum), in 14.29% of samples from horseflies (Tabanus bromius), and in one of the samples from two *Stomoxys calcitrans* flies. The reproductive LSD virus was isolated from organs of dead cattle and insects in the culture of LT and MDBK cells. The virus accumulated in cell cultures of LT and MDBK at the level of the third passage with titers in the range of 5.5–5.75 log 10 TCID50/cm^3^. Sequencing of the GPCR gene allowed us to identify this virus as a lumpy skin disease virus.

## Introduction

Lumpy skin disease (LSD) mainly infects cattle and is characterized by fever, lymphadenitis, edema of subcutaneous cellular tissue and viscera, cutaneous nodules (lumps), ocular discharge, and inflammation of the mucosae (Prozesky and Barnard 1982). It is a transmissible disease that is transferred by various arthropods (Chihota et al. 2003) and causes significant economic losses because of cattle exhaustion, hide damage, infertility, mastitis, and reduced milk production and up to 20% mortality is reported (Shalaby et al. 2016). LSD has been reported in other domestic species and wildlife naturally and through experimental infection (Young et al. 1970; Usadov et al. 2018; EFSA (European Food Safety Authority). 2015; Greth et al. 1992). LSD antibody has been detected in many species (Hedger and Hamblin 1983; Barnard 1997; Coetzer 2004) but these might also include reaction to other capripoxviruses (Davies 1982; Hamblin et al. 1990).

The causative agent of lumpy skin disease is a DNA-containing virus of the *Capripoxvirus* genus, Poxviridae family (Tulman et al. 2001). The *Capripoxvirus* genus consists of three members: sheep pox (SPPV), goat pox (GTPV), and lumpy skin disease (LSDV).

The lumpy skin disease virus (LSDV) was identified as the etiological agent for the condition in the 1940s rather than due to hypersensitivity to insect bites or plant poisoning as previously described (Macdonald 1931; Von Backstrom 1945). LSD was apparently confined to Africa until the 1980s when it emerged in the Middle East and Near Eastern countries (Wainwright et al. 2013). In 2014 and 2016, the disease spread to Europe and after being first registered in 2014 in Azerbaijan (Zeynalova et al. 2016) spread into Russia (EFSA (European Food Safety Authority) 2017). From there, it spread to the Northern Caucasus (Salnikov et al. 2018; Sprygin et al. 2018a, b). According to the data of the Information and Analytical Department of the Rosselkhoznadzor in the Russian Federation, during years 2016–2017, LSD outbreaks were registered in the Republic of Dagestan, in the Republic of in Bashkortostan, as well as on the Kazakhstan neighboring territories (Volgograd, Saratov, Samara, Astrakhan and Orenburg regions) (Rosselkhoznadzor 2017).

LSD emergence in northwestern Kazakhstan is likely to have serious consequences. The livestock industry is re-emerging including the legalization of pastoral livestock livelihoods once banned during the Soviet period. In addition, the region is inhabited by significant numbers of potentially susceptible wild animals including the critically endangered saiga antelope (*Saiga tatarica tatarica*), which Kazakhstan protects, and this population constitutes the major part of the remaining global population of this species. Various species of endemic insects and ticks occur in the steppe which might constitute a permanent vector, while the wide temperature variations may restrict the potential for vector competence and completion of the life cycles of the virus in this region.

Our investigation aimed at clarifying the cause of a disease outbreak in animals in the Atyrau region in 2016, as well as on presumptive diagnosis of LSD assessing the level of infection among various species of insects and ticks in an outbreak area. In this article, we describe the first confirmed cases of LSD in Kazakhstan in 2016, earlier reported as LSD based on clinical evidence alone to the OIE (WAHIS 2021). The paper reports in full the diagnostic and control methods used and preventive measures taken to reduce further spread of LSD in the country

## Materials and methods

### LSD affected areas and cattle populations

**Fig. 1:**
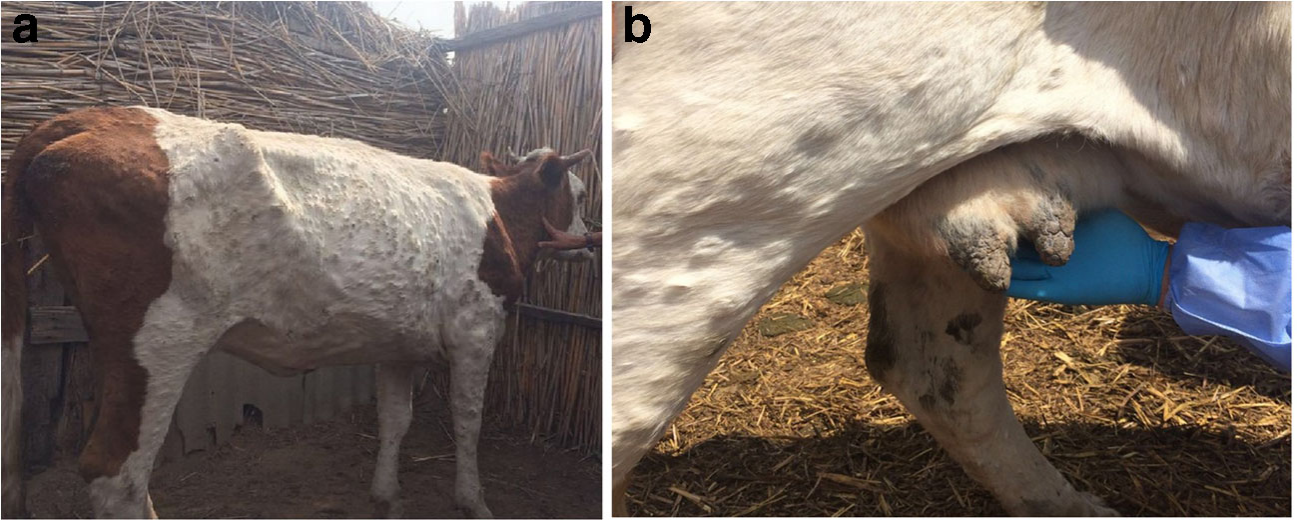
Clinical signs of lumpy skin disease

In late June and early July 2016, an outbreak of a disease of unknown etiology was observed in the village Makash in the Kurmaganzinsky district of the Atyrau region, located 50 km from the border with Russia. The first cases of the disease reported on June 30, 2016, in 5 animals in the village of Makash. The animals showed depression, inappetence, fever of 40 °C, nodule formation on the skin in various parts of the body ranging in size from 0.5 to 3 cm, serous discharge from the eyes, and increased salivation (Fig. 1). Reports of an unknown disease in the rural district continued. As of July 7, 2016, clinical signs of the unknown disease were detected in 78 cattle from the Makash rural district, and by July 21, 2016, when the disease was notified to the OIE, 459 cattle had fallen ill and 34 died (disease prevalence was 12.9% and mortality 0.96%).

In the Kurmangazinsky district, all the livestock is privately owned by citizens who keep animals in 7 livestock enterprises and several peasant farms. The farm and pasture of peasant farms are located separately from the settlements and each of them has more than a hundred heads of cattle. In total, in 2016 Kurmangazinsky district of Atyrau Province recorded 49,000 cattle, kept in 1,042 yards and farms 3,557 cattle were kept in 69 yards and farms of the Makash rural district. All settlements affected with clinical lumpy skin disease concentrated in the basin and flood plain of the Volga River running to the Caspian Sea. Located in a densely populated region along the highway and railway from Atyrau towards the Russian Federation. The Kurmangazinsky district of the Atyrau Province in the west borders on the Krasnoyarsk district of the Astrakhan Province of Russia, in the north with the Bokeyordinsky district of the West Kazakhstan region, and in the east with the Isatai district of the Atyrau Province. The territory of the Kurmangazinsky district is 20.8 thousand square kilometers, which is crossed by many rivers; the largest is the Kigach River.

### Sampling and preparation of tissues for virus detection

The Research Institute for Biological Safety Problems (RIBSP) sent an investigative mission to the region to further research the disease and samples were collected in July 2016 at a farm of the Kurmaganzy district of Atyrau Province during an outbreak of a disease with unknown etiology.

Biological samples from animals with clinical signs of the disease were also collected by state veterinarians as part of routine epidemiological surveillance. RIBSP investigators attended at the sampling. For studies, blood samples were taken from five sick cows and nine samples of internal organs (lymph nodes, spleen, lungs, skin with nodular lesions) from two dead cows. In addition to this, 13 ticks were collected from infected cattle and 21 horseflies and two biting flies were collected in the infection locus using Vavoua traps (Mihok et al. 1995). Detailed information on the collected specimens is shown in Table 1. All collected samples were preserved in liquid nitrogen and delivered to the Class III biological safety laboratory (RIBSP) in Gvardeskiy for diagnosis.

**Table 1:** The results of PCR studies and virus isolation in LT cell culture

No.	Sample	PCR tested/positive	Virus isolation					
			1 passage tested/isolated	Time of appearance of CPE (d.p.i.)	2 passage tested/isolated	Time of appearance of CPE (d.p.i.)	3 passage tested/isolated	Time of appearance of CPE (d.p.i.)
1	Lymph node	2/2	2/2	№1 (7) №2 (8)	2/2	(3)	2/2	(2)
2	Spleen	2/2	2/2	(8)	2/2	(3)	2/2	(2)
3	Lungs	2/2	2/2	(7)	2/2	(3)	2/2	(2)
4	Nodules	3/3	3/3	(7)	3/3	(2)	3/3	(2)
5	Blood of sick cattle	5/5	Н.и.	–	Н.и.	–	Н.и.	–
6	Ticks *Dermacentor marginatus*	4/4	1/0	–	1/1	№1 (6)	1/1	№1 (3)
7	Ticks *Hyalomma asiaticum* (Weiss, 1968)	9/9	2/0	–	2/1	№1 (5)	2/2	№1 (2)№2 (4)
8	Horseflies *Tabanus bromius*	21/3	1/0	–	1/1	№1 (6)	1/1	№1 (3)
9	Flies *Stomoxys* spp.	2/1	1/0	–	1/1	(7)	1/1	(3)

### Collection and preparation of samples

Preparation of samples was carried out according to the procedure described in chapter 1.1 Specimen collection, submission and preparation, OIE Terrestrial Manual 2018.

### Preparing arthropods for research

Ticks (*Dermacentor marginatus* and *Hyalomma asiaticum*), horseflies (*Tabanus bromius*), and flies (*Stomoxys calcitrans)* collected in the field were transported to the laboratory and then were washed one time in ethanol and two times in sterile phosphate-buffered saline (PBS) with (penicillin 500 IU/ml, streptomycin 1 mg/ml, mycostatin 100 IU/ml). Each insect was transferred to a small chilled porcelain mortar and ground. To prepare the suspension, 0.3 ml of PBS solution with antibiotics (penicillin and streptomycin, 100–200 IU/ml) was added. The suspension was frozen and thawed three times, and then clarified by centrifugation at 600*g* for 10 min. The supernatant was used for the study. Pools were prepared from several individuals of the same arthropod species for virus isolation.

### Virus isolation in cell culture

Virus isolation was performed as described in chapter 1.2 Virus isolation in cell culture, OIE Terrestrial Manual 2018. Primary lamb testicle (LT) cell culture and MDBK cell line were used. One ml of clarified supernatant or blood was inoculated to a confluent monolayer in a 25 cm^2^ culture flask at 37 °C and allowed to adsorb for 1 h. The culture was washed with warm PBS and filled with 10 ml of antibiotic-containing DMEM medium (penicillin 100 IU/ ml, streptomycin 0.1 mg/ml) and 2% fetal calf serum.

The flasks were checked daily during 7–10 days for cytopathic effects (CPE). If no CPE was observed by day 10, the culture was frozen three times, and the clarified supernatant was inoculated into fresh LT or MDBK culture. The specificity of the CPE was confirmed by PCR.

### DNA extraction

DNA was extracted from supernatant, blood, and culture suspension using the “DNeasy® Blood & Tissue Kit (250)”, QIAGEN, following the manufacturer’s protocol.

### PCR implementation

PCR was performed to confirm the presence of LSDV specific nucleic acid using a pair of primers: forward primer, 5′- TTTCCTGATTTTTCTTACTAT-3′ and reverse primer, 5′-AAATTATATACG TAAATAAC-3′ (El-Nahas et al. 2011). The reaction mixture contained 25 μL 2× AmpliTaq Gold (Applied Biosystems) buffers, 1 μL each of 20-pM primers, 1 μL of DNA, and up to 50 μL of water. Amplification modes were as follows: initial denaturation at 95 °С for 5 min, denaturation at 94 °С for 45 s, annealing at 56–60 °С for 45 s, replication at 72 °С for 2 min, 35 cycles, post-replication at 72 °С for 10 min. The size of the resulted amplificons was in line with the expected size of the PCR product (192 bp). The museum strain “Cattle/1986” deposited into the Microbial Collection of the RIBSP served as a positive control.

A second PCR was carried out on all positive samples to amplify the GPCR gene for phylogenetic analysis. This was done using primers designed by Le Goff et al. 2009 (Le Goff et al. 2009).

The DNA amplification of the GPCR gene was performed in a 25 μL 2× AmpliTaq Gold buffers, 1 μL each of 10-pM primers, 1 μL of DNA, and up to 50 μL of water. The PCR amplification of the GPCR gene involved an initial denaturation at 96 °C for 5 min followed by 35 cycles of final denaturation at 95 °C for 30 s, annealing at 50 °C for the 30 s, and extension at 72 °C for 30 s as previously described.

### Sequencing

Sequencing was conducted by dideoxysequencing with the use of chain-terminating dideoxynucleotides (Sanger technique) in a 16-channel sequencer Genetic Analyzer 3130 xl, Applied Biosystems (USA). POP-7 was used as a polymer for capillaries. The terminating DNA products were generated by the method of cyclic sequencing.

The evolutionary history was inferred using the neighbor-joining method (Saitou and Nei 1987). The percentage of replicate trees in which the associated taxa clustered together in the bootstrap test (1000 replicates) are shown next to the branches (Felsenstein 1985). The tree is drawn to scale, with branch lengths in the same units as those of the evolutionary distances used to infer the phylogenetic tree. The evolutionary distances were computed using the Tamura-Nei method (Tamura and Nei 1993) and are in the units of the number of base substitutions per site. Evolutionary analyses were conducted in MEGA7 (Kumar et al. 2016). The analysis involved 32 nucleotide sequences.

### Electron microscopy

To study the morphology of the LSDV the specimens of the pathology material (skin, lumps) from cattle and insects were examined. LSDV preparations were assayed in the electron microscope (JEM 100 B) with negative contrasting by 4% solution of phosphate-tungstic acid, pH 6.8.

## Results

Biological samples from sick and dead animals, ticks and insects in the disease locus were assayed for LSDV. The capripoxvirus DNA was detected by PCR in all tested samples of organs from dead animals and ticks (*Dermacentor marginatus* and *Hyalomma asiaticum*), in three specimens (14.29%) from horseflies (*Tabanus bromius)*, and in one of the specimens from two flies (*Stomoxys calcitrans*) (Table 1).

Positive specimens were used to infect the primary trypsinized lamb testicle cell cultures (LT) and the continuous bovine kidney cell line (MDBK). Both infected cell cultures demonstrated the presence of the cytopathogenic agent.

A cytopathic effect was detected at the first passage level in LT cell culture after infection with a suspension from all organs (LN, spleen, lungs, nodules) taken from dead animals.

CPE in cell culture infected with a suspension of *Dermacentor marginatus*, horseflies, and flies (*Stomoxys* spp.) was observed from the 2 passage level after 6–7 days of cultivation. During infection, the same cell culture with suspensions of *Hyalomma asiaticum* ticks, only one sample of two used, had a cytopathic effect in the second passage, and the second sample in the third passage. The cytopathogenic effect of the virus in the first passage level usually appeared within 7 to 9 days after infection. In the subsequent passages, these terms were reduced, and at the third passage level, CPE was noted for 2–4 days. Distinct CPE with 75–90% affection of the cellular layer was demonstrated in these cultures on the third passage level on the 8th day of cultivation.

Suspensions of the internal organs taken from animals post mortem were used for infecting the MDBK cell culture. The cytopathic effect of the virus in this cell culture was detected in the second passage level after 6–7 days of incubation. In the third passage, the timing of the onset of CPE was reduced and noted on the third day with the defeat of 70–80% of the monolayer cells.

The virus accumulated in these cultures (LT, MDBK) on the third passage level in titers within 5.5–5.75 log 10 TCID_50_/cm^3^.

The specificity of the isolated viruses was confirmed in PCR and by electron microscopy. In all specimens, an amplification product sized 192 bp typical for the LSDV was generated. The study of the morphology and fine structure of the negatively stained preparations showed viral particles typical for LSDV. The size of the virions varied within 300 × 370 nm, while the bulk mass of particles (90%) was sized 300 × 350 nm.

A virus isolated from the skin nodules of a dead animal was selected for archiving “Nodulares/Dermatitis/Atyrau-2016” and deposited into the Microbial Collection of the RGE RIBSP, and later this same virus was used to produce an attenuated candidate vaccine strain.

Sequencing of the GPCR gene allowed us to identify this virus as a lumpy skin disease virus.

### Disease control

After the first cases of the disease, in the Kurmangazy region, the veterinary service introduced restrictive measures to localize and eliminate the disease. The movement of all species of domestic animals was prohibited, and a daily farm visit was carried out in order to identify animals with clinical signs of lumpy skin disease. After laboratory confirmation of the disease, the affected area (Kurmangazy region) was quarantined. All 459 animals with clinical signs were culled and the owners were compensated at the market value of the animal. Disinfection of affected sites was completed including 28.116 m^2^ of land. The remaining live animals were treated with repellents.

After confirming the diagnosis and reporting the disease to OIE, all the cattle of the Atyrau Province were vaccinated, as well as animals in the risk zone (threatened zone) of the West Kazakhstan and Aktobe Province. Vaccination was carried out using the lumpivax® vaccine manufactured by the Kenya Veterinary Vaccine Manufacturing Institute (KEVEVAPI).

## Discussion

The lumpy skin disease virus is considered a serious pathogen of transboundary significance (Saegerman et al. 2018). Since about 2015, the disease has spread from African countries through the Near East into Europe, Azerbaijan, and Russia, and caused significant economic losses (Zeynalova et al. 2016; Salnikov et al. 2018; Sprygin et al. 2018a, b; Saegerman et al. 2018). The disease was previously recorded only in tropical and sub-tropical countries. There remain many gaps in knowledge on the establishment of LSD in temperate latitudes, and what role rising environmental temperatures, mediated through vectors, will play in LSD epidemiology. The first cases of the disease were reported in Russia in 2015 and this led to studies regarding potential arthropod carriers of LSDV. Virus DNA was found in 13 species of ixodid ticks (Gazimagomedov et al. 2017), and in 2017, DNA of the virus was detected in *Musca domestica L.* (Sprygin et al. 2018a, b). At the same time, the infection prevalence of some of the studied species of ticks reached up to 16.3% in the epidemic zone (Gazimagomedov et al. 2017).

Disease in Kazakhstan was first reported in early July 2016 in the Kurmangazy district of Atyrau Province. LSD diagnosis from this outbreak is confirmed by the laboratory assays (PCR) of the samples from sick and dead animals, as well as confirmation of the presence of the virus in the arthropods collected in the epidemic focus. The etiological agent was isolated in the primary lamb testicle cell culture and in the continuous cell culture MDBK. Further sequencing of the gene GPCR and the phylogenetic analysis showed the close genetic relationship of the isolated capripoxvirus with a group of LSD viruses including those circulating in the areas of the Russian Federation adjacent to Kazakhstan. Emergence of the disease in the western regions of Kazakhstan from probable transboundary sources was confirmed. According to the information in Russia during May–June 2016, there were 163 LSD suspected foci including three in the Astrakhan region bordering the Atyrau Province of Kazakhstan (Rosselkhoznadzor 2017). Until this time, LSD was considered exotic to Kazakhstan and measures were not taken to prevent or diagnose the disease routinely. Through prompt action, the disease was apparently eliminated through stamping out policy in the epidemic focus in Kazakhstan, and a strategy was developed to prevent further incursions from infected countries. The control strategy is based on vaccination of the entire susceptible livestock in Kazakhstan using lumpivax® vaccine manufactured by Kenya Veterinary Vaccines Production Institute (KEVEVAPI).

The peak activity of blood-sucking insects and ticks in Kazakhstan falls in April and October (Faulde n.d.). The emergence of the disease was coincident with a peak of vector activity, and introduction of LSDV on to the territory of Kazakhstan was likely either through the movement of infected livestock and subsequent transmission of the virus by blood-sucking insects, as reported elsewhere, or through movements of vectors, although the latter is less likely as vector movements are over relatively small distances, flies and mosquitos mostly only a few hundred meters unless wind assisted [32, 36]. The transmission pathways and epidemiology of LSD are as yet not fully understood. LSDV has been detected in biting flies (*Stomoxys* and *Musca* spp), *Aedes* mosquitoes, in ticks (*Rhipicephalus appendiculatis*), (*Amblyomma hebraeum*), and (*Rhipicephalus (Boophilus) decolaratus*) (Tuppurainen and Oura 2011; Chihota et al. 2001; Last 2017; Issimov et al. 2020). Alexandrov (Alexandrov 2017) reported possible mechanical transmission of the LSD virus by *Tabanus spodopterus* females during an outbreak in 2016 in Bulgaria (Alexandrov 2017). Later, Sohier et al. (2019) experimentally demonstrated that *Haematopota* spp. (horseflies) can mechanically transmit the LSD virus (Sohier et al. 2019). In our study, several arthropod species including ixodid ticks (*Dermacentor marginatus* and *Hyalomma asiaticum)*, horseflies (*Tabanus bromius*), and other biting flies (*Stomoxys calcitrans*) collected in the disease focus were assayed as potential transmitters. All tick samples were positive, and a proportion of horse flies and *Stomoxys* flies (Table 1). The first LSDV isolate in cell culture was obtained from sampled horseflies (*Tabanus bromius*) collected during the outbreak of the disease. These results support the studies of Sohier et al. (2019), which showed experimentally that horseflies can mechanically transmit LSDV. The mechanical transmission of LSDV by ixodid ticks has been shown by many researchers, but until now, there has been little information on the species of ticks and arthropods which might play a role in the transmission of LSDV. Our studies have shown that all individuals sampled, of both species of ticks collected from the region of the outbreak of the disease, were PCR positive for LSDV, and the virus was isolated from the pool of ticks of both species, through cell culture. The ticks and biting flies recorded here are widespread throughout western Kazakhstan and could become vectors of mechanical transmission of the LSDV among domestic and wild animals if the virus is established in the animal population. This opportunistic study contributes to published data on the vector transmission of the disease (Tuppurainen et al. 2011). The persistence of the virus in the region will depend on the completion of life cycles of LSD across a zone with an extreme climate and this possibility needs to be monitored through appropriate surveillance annually.

Studies have shown that a novel cattle disease in the Atyrau Province of Kazakhstan in 2015 was caused by infection with the lumpy skin disease virus. Virus was also detected among arthropod horseflies, biting houseflies, and or ticks suggesting the possibility of these species as vectors of LSD in this region.

## Data Availability

All data needed to evaluate the conclusions in the paper are present in the paper. Additional data related to this paper may be requested from the authors.
